# Distinct Effects of Royal Jelly on Human Endothelial Cells Under High Glucose Condition 

**Published:** 2018

**Authors:** Omid Cheraghi, Mina Abdollahpourasl, Aysa Rezabakhsh, Reza Rahbarghazi

**Affiliations:** a *Department of Biology, Faculty of Natural Sciences, University of Tabriz, Tabriz, Iran.*; b *Stem Cell Research Center, Tabriz University of Medical Sciences, Tabriz, Iran.*; c *Department of Biology, Faculty of Science, Urmia University, West Azerbaijan, Iran. *; d *Department of Pharmacology and Toxicology, Faculty of Pharmacy, Tabriz University of Medical Sciences, Tabriz, Iran. *

**Keywords:** Royal jelly, HUVECs, Stemness feature, Fatty acids profile, Autophagy, MMPs activity

## Abstract

To assess different effects of royal Jelly in protecting the human endothelial cells from high glucose level, human umbilical vein endothelial cells were exposed to various concentrations of royal jelly, from 0.625 to 10 mg/mL, at the presence of 5 and 30 mM glucose contents over a course of 72 h. In addition to cell viability assessment by conventional MTT assay, we also analyzed the feature of stemness by expression of Sox-2 and CD133 factors. Moreover, fatty acid profile, the expression of autophagy-related factor, namely microtubule-associated protein light chain 3 and activity of metalloproteinase 2 and 9 and were investigated. Royal jelly supplementation at the concentrations lower than 2.5 mg/mL did not influence the survival rate of cells and partially blunted the cytotoxic effects of 30 mM glucose. The expression of CD133 and Sox-2 factors were increased by royal jelly alone. Interestingly, an up-regulated expression of Sox-2 (58.8 ± 4%) coincided with a reduction in the levels of CD133 (15.1 ± 8.3%) in the combined treatment. We notified that the contents of palmitate and trans-palmitate as well as linoleate decreased by 30 mM glucose content while cis-palmitate levels increased when RJ returned them to near-normal levels (*p *< 0.05). The expression of autophagy marker was prominently induced in the presence of royal jelly in both conditions (*p *< 0.05). The glucose-induced activity of metalloproteinases was also reduced. Royal jelly is able to attenuate the abnormal status of 30 mM glucose condition in endothelial cells by different mechanisms.

## Introduction

Endothelium shows a critical role in the regulation of angiogenesis during tissue healing or transplantation. A wealth of data implies that numerous condition accounts for the aberrant pathological angiogenesis which is governed mainly by endothelial insult or the loss of normal function, notably during the chronic exposure to diabetic condition (1, 2). These endothelial dysfunction couples with an impairment of the capacity of the endothelium to maintain vascular homeostasis as a result of fundamental change in flux of glucose via polyol pathway, intracellular formation of reactive oxygen species, accumulation of advanced glycation end-products (AGEs), etc. in endothelial cells (ECs) (3, 4). These conditions contributed to dysregulated angiogenesis, resulting in uncontrolled de novo vascularization and the development of abnormal status (5). 

It is known that several natural products such as a honey bee Royal jelly (RJ) could be used as a therapeutic agent due to presence of natural components. RJ, a milky secretion produced by hypo-pharyngeal and mandibular glands of worker honey bees (*Apis millifera*), is being widely used with various noticeable advantages today (6). Following chemical composition analysis, it has been elucidated that RJ encompassed crude proteins (12.5%), sugars (11%), lipids (5%), vitamins, and free amino acids (7). Additionally, many trace minerals, different enzymes, active antibacterial and antibiotic ingredients, pantothenic acid (vitamin B5), vitamin B6 (pyridoxine) and trivial amounts of vitamin C, but none of the fat-soluble vitamins such as A, D, E, and K have been proved (7). 

A plethora of experimental studies unveiled that pharmacological concentration of RJ exerted a vast array of activities, including vasodilatory, anti-inflammatory, and immune-modulatory effects (8). For example, major RJ protein 3, a 70 kD glycoprotein, could potently suppress the production of interleukin-4, 2 and IFN-γ (9). Vitteck el al. previously acclaimed the positive modulatory effects of RJ on lipid metabolism and experimental induced atherosclerosis (10). They showed hypolipidemic effects of RJ by the diminishing serum and hepatic cholesterol and fat storage in the liver. It also well-determined that 10-Hydroxy-2-decenoic acid (10-HDA), a major fatty acid component of RJ, actually prohibited *in-vitro* tubulogenesis induced by Vascular Endothelial Growth Factor (VEGF) (7). Among the advantages proposed for RJ, a potent antioxidant activity is documented on account of phenolic compounds and flavonoids (11). Delkhoshe-Kasmaie *et al*. acclaimed that RJ potently attenuated taxol-induced testicular damages in rats by the modulation of antioxidant status and E2F1 transcription factor (12). 

We hypothesized here, that the presence of RJ could diminish the adverse effects of high glucose condition (HGC) on ECs over a period of 72 h. Therefore, the modulatory effects of RJ were scrutinized in terms of ECs viability, fatty acids components, stemness state, metalloproteinase (MMP) activity, and autophagy status. 

## Experimental

*Cell culture*

To perform the current study, we used Human Umbilical Vein Endothelial Cells (HUVECs), as extensively used in *in-vitro* condition for investigation of ECs function and pathology. HUVECs were purchased from National Cell Bank of Iran (Pasteur institute, code: C554) and routinely grown in Dulbecco’s modified Eagle’s medium/F12 (DMEM/F12; GIBCO-Invitrogen) containing 10% Fetal Bovine Serum (FBS, GIBCO), 100 U/mL penicillin- 100 μg/mL streptomycin (Biosera) and maintained at 37 °C and 7% CO_2_. The exhausted medium was replaced every 3-4 days. For the subculture of HUVECs, Cells grown to 70–80 % confluence were rinsed and detached by using 0.25% Trypsin-EDTA solution (GIBCO). In our study, third passage of cells was subjected to all experiments. 

*MTT assay*

To determine whether or not RJ could reverse the adverse effects of HGC on HUVECs viability rate, we used a conventional MTT assay. Briefly, HUVECs were seeded at initial density of 2 × 10^4^ cell per each well of 96-well plates (SPL). After reaching to 70-80% confluency, cells were exposed to various concentration of RJ including 0.625, 1.25, 2.5, 5 and 10 mg/mL in DMEM/F12 medium supplemented with 2% FBS and concurrently co-treated with 5 mM and 30 mM glucose levels over a course of 72 h. Next, the supernatant was discarded and replaced with 200 μL MTT (1-(4, 5-dimethylthiazol-2-yl)-3, 5-diphenylformazan) solution (5 mg/mL) and subsequently kept for 4 h at 37 °C. To dissolved formazan crystals, MTT solution was further removed and 100 μL of Dimethyl Sulfoxide (DMSO, Merck) was added per well. The absorbance was recorded at 570 nm using a microplate reader system (BioTek, USA). Finally, the cell viability rate was expressed as % of control. Three independent set of experiments were provided in octuplicate. 

*Quantification of CD133 and Sox-2 positive cells under the co-incubation of RJ and HGC*

The possible stimulating/inhibitory effect of RJ, alone or in combination with HGC, on the percentage of cells expressing Sox-2 and CD133 was revealed by using FACSCalibur system (BD Bioscience). In short, a number of 1 × 10^5 ^cells were plated in each well of 24-well plates and subjected to four different culture conditions. Protocol I: 5 mM glucose; Protocol II: 30 mM glucose; Protocol III: 5 mM glucose- 2.5 mg/mL RJ and Protocol IV: 30 mM glucose- 2.5 mg/mL RJ co-treatment. By 72-h of incubation period, cells were detached and washed twice with PBS solution. A 1% bovine serum albumin solution (BSA; Sigma) were used to blocked cells through a period of 20 min. Next, a group of antibodies directed against CD133 (FITC-conjugated mouse anti-human CD133; Miltenyi Biotec) and Sox-2 (FITC-conjugated mouse anti-human Sox-2; Millipore) were used according to manufacturer’s recommendation. To subtract background staining, the cells were stained with appropriate isotype-matched antibodies. The obtained results were calculated by FlowJo software ver.7.6.1. 

*Total Fatty Acids profile analysis by gas chromatography*

For total fatty acid profile analysis, a direct trans-esterification assay was carried out as previously described (13). At least, 1 × 10^5^ cells from four different groups, control (5 mM), 30 mM, and cells co-incubated with 2.5 mg/mL RJ under normal and HGC, were trans-esterified to methyl esters by the addition of 200 µL acetyl chloride reagents in 2 mL methanol-hexane solution and methanolyzed at 100 °C for 1 h. Thereafter, 5 mL of 6% K_2_CO_3_ solution was added for nullifying the mixture. The superior phase was then collected for further assessment. The patterns of generated methyl esters were revealed by a gas chromatography (GC) system (Model 610, Buck Scientific), normalized to an internal standard and calculated using Peak Simple software ver. 3.59 (SRI Inc.). The outcome values for a panel of saturated, monounsaturated and polyunsaturated fatty acids, namely Palmitate (C16:0), Trans-palmitoleate (trans-C16:1), Cis-palmitoleate (cis-C16:1), Stearate (18:0), Oleate (C18:1), and Linoleate (C18:2) were calculated and indicated as percentage of total extracted fatty acids to get an obvious pattern. The data were representative of three independent experiments.

*Immunoblotting analysis*

Maintaining usual activity of autophagy-related markers in the various cells is touted to be crucial to homeostasis especially on the vasculature. To testify whether RJ could or not act directly on autophagy status of HUVECs under HGC, western blot analysis was performed to monitor the expression of microtubule-associated protein light chain 3 (LC3). LC3 is conceived as a key autophagy initiating marker. On day 3, the cells were detached by 0.25% Trypsin-EDTA solution and directly lysed in ice-cold lysis buffer system (Sigma-Aldrich) solution enriched with cocktail enzyme inhibitors. After sonication, the cell lysates were centrifuged at 14000 *g* for 20 min at 4 °C. Total proteins in the supernatant were determined using a NanoDrop 1000^TM^ spectrophotometer system (Thermo Scientific). A 100 µg of each sample was loaded on 12% SDS polyacrylamide gel electrophoresis followed by electrotransferring to 0.2 μm immune-Blot^TM^ Polyvinylidene Difluoride membrane (PVDF; Millipore). The membranes were probed with a rabbit anti-human LC3 monoclonal antibody (at a dilution of 1:1000; overnight, Abcam) and then with anti-rabbit IgG secondary antibody conjugated to horseradish peroxidase (at a dilution of 1:1000; 1 h, Abcam). Finally, the bands were visualized using the ECL system (Millipore) and densitometric analyses of immunoblots were conducted with ImagJ software Version 1.44p (NIH, USA). Moreover, quantification of each band was carried out by the corresponding densitometry of the β-actin (dilution: 1/1000; Abcam). This experiment was performed in triplicate.

*Gelatin zymography assay*

Levels of MMP-2 and -9 were qualitatively assessed by gelatin Zymography technique. Briefly, 100 μg of total protein from each sample was mixed with equal volumes of 2ME-free Laemmli’s sample buffer and then subjected to 12% SDS-PAGE separation, which polymerized with 0.2% gelatin. Following electrophoresis, the gels were washed twice with 2.5% Triton X100 for 1 h, subsequently incubated overnight at 37 °C in buffer containing 50 mM Tris-HCl, (pH 7.4), 5 mM CaCl_2_, and 0.02% NaN_3_. Thereafter, gel was stained with 0.2% Coomassie blue dye solution for 20 min and destained with destaining solution until white bands were confirmed on a blue background. Gels were scanned using an HP Scanjet G3110 apparatus (Hewlett-Packard Company, USA). Images were converted to black and white in Adobe Photoshop software CS5 (Middle Eastern ver.12.0×32). This experiment was run in triplicate. 

*Statistical analysis*

Outcome values depicted in the text, and Figures are expressed as mean ± SD. For multiple comparisons One-way (ANOVA) and Tukey’s test was performed by using the Instat GraphPad software *p *< 0.05 was considered significant. In histograms, statistical difference between the groups is shown by brackets with *p < 0.05, ** *p *< 0.01 and ***p < 0.001.

## Results

*RJ reduced the adverse effects of HGC on HUVECs in defined concentrations*

**Figure 1:**
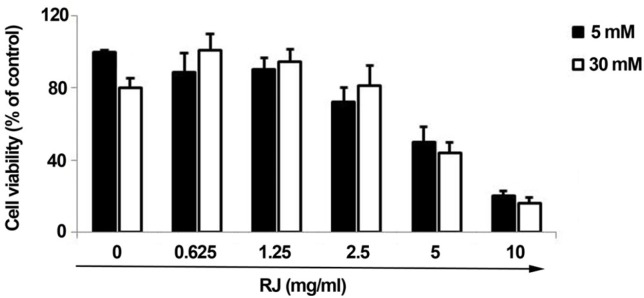
MTT assay to evaluate the effects of RJ on the viability of HUVECs over a period of 72 h. Various concentrations of RJ, from 0.625 to 10 mg/mL, were used in the absence or presence of elevated glucose contents. The data are expressed as a percentage of basal value

To investigate the role of RJ on pathological status of ECs under HGC, HUVECs were co-incubated with different concentrations of RJ in two main sets of 5 mM and 30 mM glucose levels. According to data obtained from MTT assay, the cell viability rate was reduced in cells-exposed to HGC as compared to parallel control (fewer than 80% of control). As illustrated (Figure 1), a dose-dependent reduction in the viability rate was also observed in cells being-exposed to RJ alone (Figure 1). Therefore, the single treatment of HUVECs with RJ yielded prominent cytostatic effects, especially at higher concentrations. Following examining the combined regime of RJ and HGC, it was revealed that RJ could only attenuate the HGC-derived cytotoxic effect when used at concentration range from 0.625 to 2.5 mg/mL (Figure 1). Of interest, it should be noticed when RJ is used at the concentrations more than 2.5 mg/mL, not only could not blunt HGC cytotoxicity, but also it intensifies the unfavorable conditions of highly amount of glucose. After testing HUVECs with different concentrations of RJ in two sets of glucose levels, it could be inferred that the application of RJ at higher concentrations, regardless of 30 mM glucose toxicity, induces cytotoxic effects on the EC lineage in different milieu.

*Stemness features of HUVECs under RJ treatment*

**Figure 2:**
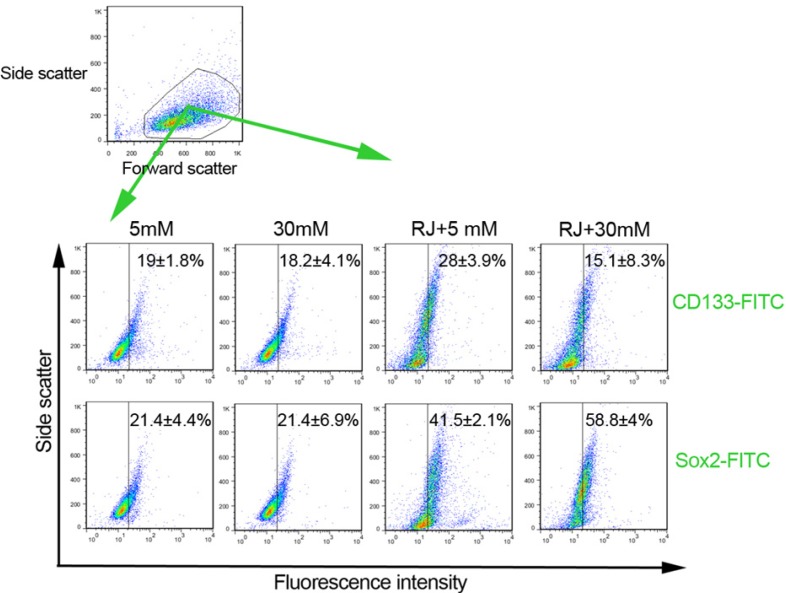
Representative illustration of the effect of RJ on features of HUVECs stemness under 5 and 30 mM glucose conditions. Both CD133 and Sox-2 factors were analyzed by using flow cytometry methods. Data are expressed as mean ± SD (n = 3).

Both Sox-2 and peculiarly CD133 factors are known to be associated with stemness properties. To see how RJ, HGC and their combination could affect stemness features of HUVECs, we intended to quantify the number of CD133 and Sox-2 factors by flow cytometry analysis (Figure 2). Based on our findings, the expression rates of the CD133 and Sox-2 inversely reduced or unchanged in cells being-exposed to HGC (Figure. 2). In contrary, we observed that the use of RJ increased the number of cells expressing CD133 factor (28 ± 3.9 versus 19 ± 1.8%) (Figure 2). The cells under RJ treatment were more prone to express higher levels of Sox-2 (41.5 ± 2.1%) as compared with normal condition (21.4 ± 4.4%). Besides RJ alone environment, the expression level of Sox-2 appeared to be increased more than 2.7 folds at the presence of RJ and 30 mM glucose as compared with untreated condition. We found a 0.7-fold drop in levels of CD133 when HUVECs were exposed to a combination of RJ and HGC. Our expression profile of stemness features confirmed an upward trend of both CD133 and Sox-2 by RJ alone while the effectiveness of RJ for acquired stemness, varying depending on the type of factor, could be increased or blunted when concurrently used with 30 mM glucose.

*Fatty acids profile was actively changed by RJ*

**Figure 3:**
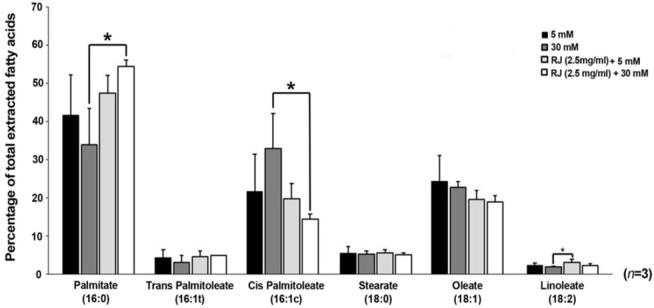
GC analysis of fatty acid composition in HUVECs treated with 2.5 mg/mL RJ in the absence of presence of 30 mM glucose. one-way (ANOVA) and Tukey’s test. *p < 0.05

A number of saturated, mono and poly-unsaturated fatty acids were monitored to see how RJ change lipid balances in HUVECs treatment under 5 and 30 mM glucose contents. According to GC analysis, fatty acid compositions, including palmitate (16:0), stearate (18:0), linoleate (C18:2), palmitoleate (both cis- and trans-C16:1), and oleate (C18:1) differed in various conditions used here (Figure 3). At the end of the 72 h incubation period, the levels of saturated palmitate diminished in HGC-treated cells were compared with all other groups (*p *_30mM versus RJ+30mM _**< **0.05), while in RJ-treated cells palmitate levels significantly increased either in the presence or absence of 30 mM glucose. The increase was higher in the simultaneous presence of glucose and RJ versus RJ alone (Figure 3). Consistent with effect of HGC on the values of palmitate, the mono-unsaturated trans-palmitoleic acid (trans-C16:1) level was also diminished in the same manner, but differences between the groups did not reach a significant level (*p *> 0.05). In contrary to trans-palmitoleic acid levels, it seems that 30 mM glucose alone, but not in combination with RJ, increased the contents of cis- palmitoleic acid as compared with others (*p *_30mM glucose versus RJ+30 mM glucose _< 0.05). Interestingly, the lowest rate of cis- palmitoleic acid was achieved whenever the combined regime of RJ and HGC introduced to HUVECs. On the subject of stearate, significant statistical changes were not observed in any groups (*p *> 0.05). The slight non-significant decrease in content of oleate (c18:1) was achieved in all treatment groups - in particular – when RJ combined with 30 mM glucose (*p *> 0.05) (Figure 3). Linoleate (C18: 2) followed a similar trend as previously indicated for palmitate. We confirmed that RJ enabled cells to synthesize linoleate (*p *_RJ versus RJ+30mM _< 0.5) while a co-incubation of HUVECs with RJ and HGC closed it to control levels.

*RJ could easily induce autophagic state under 5 mM glucose content rather than HGC*

**Figure 4:**
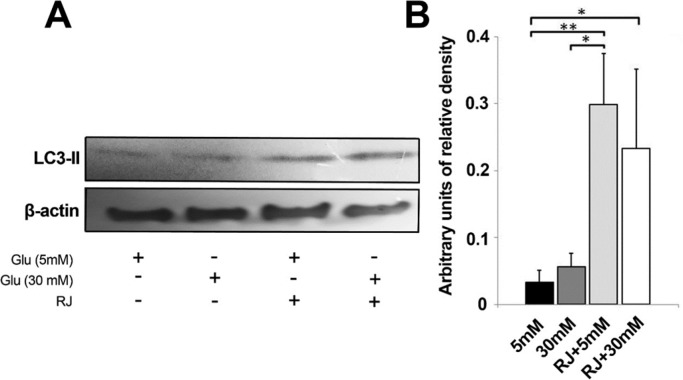
Western blot analysis of autophagy markerLC3-II after 72 h in four different conditions. This experiment was repeated three times. One-way ANOVA and Tukey’s test. *p < 0.05, ** *p *< 0.01 and ***p < 0.001

The capacity of RJ to inhibit or exacerbate autophagy status under HGC was further analyzed by western blotting technique (measured in terms of LC3-II expression) (Figure 4A-B). We showed a slight higher protein expression of the autophagy marker in 30 mM glucose environment as compared to control (Figure 4A-B). Despite lack of statistically significant differences between sample of 5 and 30 mM glucose, the 1.66 yield increase of LC3-II in 30 mM samples is, however worth considering (Figure. 4-B). The use of RJ alone (2.5 mg/mL) or in combination with 30 mM glucose content triggered a bulk increase in the expression of LC3-II 

(*p *_5mM versus RJ _< 0.01 and *p *_5mM versus RJ+30mM _< 0.05). Noticeably, RJ combined with 30 mM glucose meaningfully blunted the LC3-II expression in comparison with RJ group, only 0.78 fold change expression were obtained (*p *> 0.05). In agreement with these observations, the exposure of EC lineage to 2.5 mg/mL RJ increased LC3 expression and potently stimulated the number of cells entering autophagic state either in 5 or 30 mM glucose conditions.

*Changes in the activity of MMP-2 and MMP-9 were seen under experimental procedure*

**Figure 5:**
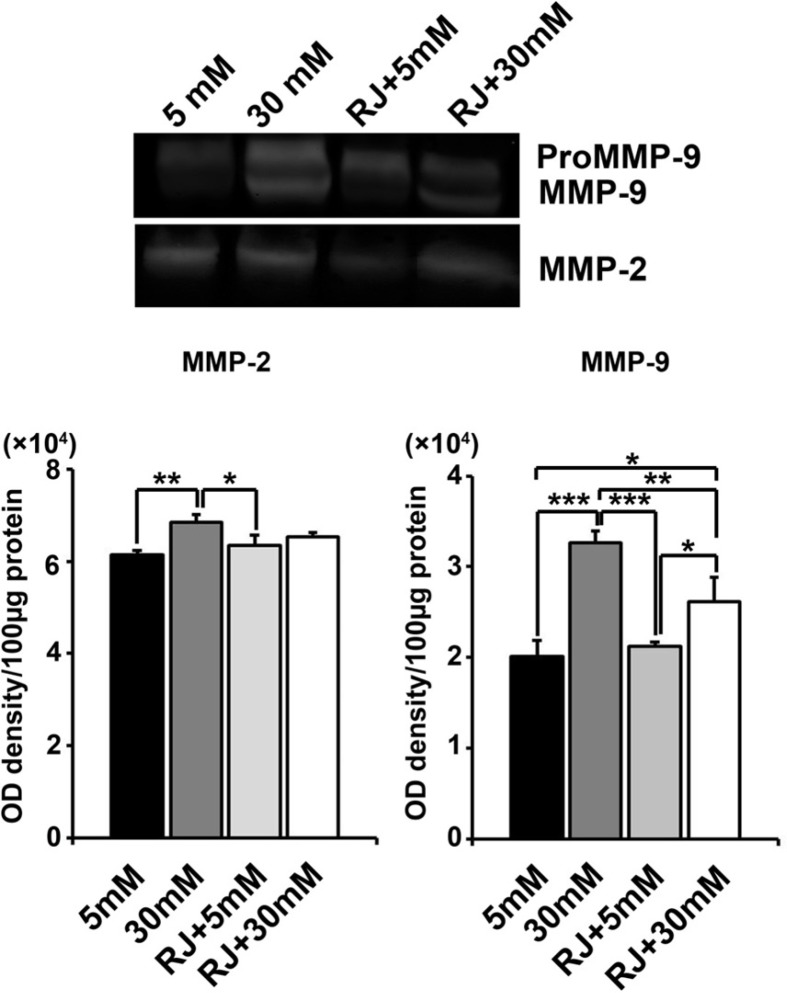
Effect of RJ on 30 mM glucose-induced MMP-2 and -9 activities after 72 h. (A) HUVECs were incubated simultaneously with RJ (2.5 mg/mL) and elevated glucose contents for 72 h. The cell lysates were examined for the gelatinolytic activity of MMP-2 and -9 by gelatinous zymogram. One-way ANOVA and Tukey’s test. *p.<.0.05, ** *p.*<.0.01 and ***p < 0.001

Zymographic analysis revealed a steady increase in the activity of HUVECs MMP-2 and -9 under HGC as compared with normal condition (MMP-2: *p *_5mM versus 30mM_ < 0.01; MMP-9: *p *_5mM versus 30mM_ < 0.001) (Figure 5). No obvious changes were obtained in MMPs activity of cells-being exposed to RJ alone (*p *_5mM versus RJ _> 0.05). Simultaneous use of RJ and 30 mM glucose caused a declined in the activity of both MMP-2 and -9 when compared to 30 mM-treated control (MMP-2: *p *_30mM+RJ versus 30mM_ > 0.05; MMP-9: *p *_30mM+RJ versus 30mM_ < 0.001) and returned to near controls level (Figure 5). We showed that RJ could not alter the gelatinolytic activity of HUVECs under normal condition; however, it potently neutralized the aberrant activity of MMPs in HGC. 

## Discussion

It is believed that a chronic disturbance in endocrine homeostasis of carbohydrate metabolism occurred particularly in diabetic condition, and ECs are substantially in the frontline against these chaotic conditions (1,2). Today, herbalists discovered that RJ could be used in human medicine due to an increased state of body’s natural resistance to different conditions (14). Our present experiment sought to pinpoint the effect of RJ on distinct EC parameters like viability, stemness state, metalloproteinase (MMP) activity and autophagy status related to normal and HGC. 

In the present study, we notified that RJ was a potent cytotoxic agent for HUVECs either in 5 and 30 mM glucose levels when used in higher concentration. Nevertheless, low doses of RJ probably can suppress the cytostatic effects of 30 mM over a period of 72 h. It was previously shown that 10HDA, a major fatty acid component of RJ, could inhibit EC proliferation, migration and *in-vitro* tubulogenesis responses (7). 

Our experiment has identified CD133^+^ and Sox-2^+^ cells in HUVEC cultures and presented an *in-vitro* model for the study of stemness features in different conditions. We showed in normal and abnormal glucose contents striking CD133 and Sox-2 staining of HUVECs at the presence of 2.5 mg/mL RJ. As far as we understand, anyone did not provide a detailed statement about the effect of RJ on stemness features under HGC. Only, a recent study reported different impacts of plural components of RJ on neurogenesis (15). On the one hand, it was acclaimed that the extract of RJ caused an enhanced phosphorylation of extracellular signal-regulated kinase 1 or 2 (ERK1/2) and cAMP-response element-binding protein in neural stem cells, which facilitated differentiation of all types of brain cells. On the other hand, 10-HDA alone accelerated neurogenesis while inhibited astrocyte differentiation rate (15). Therefore, the difference in the performance of RJ could be related to the concentrations of different components or extract type examined in various cells. Our finding suggested contradictory trends in CD133 and Sox-2 expression by RJ under HGC. Although, the exact mechanism of RJ in acquiring stemness properties during HGC was not addressed in our experiment, we imagine that the proposed dual effect of RJ on both conditions can be presumably mirrored for *in-vivo* milieu.

Biosynthesis of mono-unsaturated fatty acids, oleate and palmitoleate, are under control of a microsomal enzyme named stearoyl-CoA desaturase (SCD) activity, from saturated lipids such as stearate and palmitate, respectively (16). Regarding fatty acids analysis, the levels of palmitate (16:0) and trans-palmitoleic acid (trans-C16:1) fatty acids were reduced while cis-palmitoleic acid (cis-C16:1) value increased under HGC. Under high-carbohydrate diet, uncontrolled activity of SCD resulted in an improper ratio of saturated to mono-unsaturated which in turn affects cell membrane fluidity (17). Hence, we speculate that the single use of RJ or in combination with 30 mM glucose mitigates the adverse effect of high amounts of glucose by the regulation of SCD activity, even less than normal range. Our study of groups of cells undergoing RJ alone or in combination with 30 mM glucose gives valuable insights into functionality of RJ in attenuating the activity of SCD.

We further discovered an enhanced autophagy activity by the expression of LC3-II under HGC and especially RJ containing environment (with or without 30 mM glucose). We strongly confirmed an outstanding change in basal autophagic status when cells incubated with RJ. Based on numerous studies, autophagy functions as a protective machinery to prevent HGC-mediated cell death by maintaining energy homeostasis (18). For example, Xu *et al*. acclaimed that the activation of autophagy was to protect renal tubular cells from lipotoxicity under HGC and contributing to the clearance of highly insoluble aggregates (19). In line with this statement, a marked up-regulation of LC3-II by RJ could presumably be an appropriate strategy to reduce HGC-mediated cell cytotoxicity peculiarly in ECs. However, the precise mechanisms underlying in the regulation of autophagy by RJ necessitate gaining considerable momentum. 

It was reported that elevated contents of circulating glucose *in-vivo *changed MMP activity in various cell types, especially ECs (20). Hence, gelatinous zymogram assay was performed to understand the possible role of RJ on the cellular origin of MMP-2 and 9 activities in vascular cells under 30 mM glucose condition. The elevation in HUVECs gelatinase activity MMP-2 and prominently MMP-9 under 30 mM glucose was reflected by changes in the plasma of diabetic subjects (20). Although RJ potently diminished the MMPs activity in HUVECs at both conditions, but a slight increase in gelatinolytic activity of RJ at both conditions was achieved as compared with untreated cells. Majtan and colleagues already detected not statistical significant stimulatory effects of RJ on cutaneous cells, but they found an upward trend in mRNA expression of MMP-9 (21). On the other hand, it was reported that 10HDA attenuated VEGF-associated angiogenesis, to some extent, due to inhibition of MMPs in ECs (7). Concurrently, HUVECs treated with RJ in this study showed significantly decreased gelatinolytic activity either under normal and HGC. Due to critical role and pro-inflammatory action of MMP-9 (22), it is reasonable to reveal the more pronounced effects of RJ on MMP-9 compared to MMP-2. We, therefore, notified that RJ could attenuate MMPs malfunction and deregulation of extracellular the matrix which may be initiated by HGC. 

Our results show that the *in-vitro* behavior of ECs, cultured on HGC, can be affected by the presence of RJ. The differential stemness feature, regulatory mechanisms on lipid profile, extracellular migration rate, and autophagy status are generally modulated by RJ. Furthermore, investigations of the different characteristics of various RJ components will augment new avenues for deciphering the molecular mechanisms of angiogenesis under diabetic condition.
